# Greater lateral root branching density in maize improves phosphorus acquisition from low phosphorus soil

**DOI:** 10.1093/jxb/ery252

**Published:** 2018-07-26

**Authors:** Xucun Jia, Peng Liu, Jonathan P Lynch

**Affiliations:** 1 College of Agronomy, Shandong Agricultural University/State Key Laboratory of Crop Biology, Tai’an, Shandong Province, China; 2 Department of Plant Science, The Pennsylvania State University, University Park, PA, USA

**Keywords:** Branching density, lateral root, maize (*Zea mays*), phosphorus, root architecture, topsoil foraging

## Abstract

The development of crops with better growth under suboptimal phosphorus availability would improve food security in developing countries while reducing environmental pollution in developed countries. We tested the hypothesis that maize (*Zea mays*) phenotypes with greater lateral root branching density have greater phosphorus acquisition from low phosphorus soils. Recombinant inbred lines with either ‘many short’ (MS) or ‘few long’ (FL) lateral root phenotypes were grown under high and low phosphorus conditions in greenhouse mesocosms and in the field. Under low phosphorus in mesocosms, lines with the MS phenotype had 89% greater phosphorus acquisition and 48% more shoot biomass than FL lines. Under low phosphorus in the field, MS lines had 16% shallower rooting depth (D_95_), 81% greater root length density in the top 20 cm of the soil, 49% greater shoot phosphorus content, 12% greater leaf photosynthesis, 19% greater shoot biomass, and 14% greater grain yield than FL lines. These results are consistent with the hypothesis that the phenotype of many, shorter lateral roots improves phosphorus acquisition under low phosphorus availability and merits consideration for genetic improvement of phosphorus efficiency in maize and other crops.

## Teaser

Maize genotypes with greater lateral root branching density have superior phosphorus uptake, growth, and yield in low phosphorus soil.

## Introduction

The need to sustain a growing human population with a degrading natural resource base is a paramount challenge of the 21st century (Cordell *et al*, 2009; Godfray *et al.*, 2010). Phosphorus (P) is frequently a limiting element for plant growth and development. In the low-input agriculture characteristic of developing nations, low P availability is a primary constraint to food production and economic development. Intensive fertilization in high-input agriculture causes massive environmental pollution. Therefore, the improvement of P acquisition and use by crop plants is critical for economic, humanitarian, and environmental reasons (Vance, *et al.*, 2003; Lynch, 2007, 2011; Withers *et al.*, 2014; Cordell and White, 2015).

Plants have evolved several strategies for P acquisition and use in low P environments, including efficient P utilization and enhanced acquisition (Lynch and Brown, 2001; Vance, 2001; Lambers *et al.*, 2013; Miguel *et al.*, 2015). Roots play a key role in P acquisition because of spatial variation in soil P availability resulting from its low mobility, and factors related to P availability, such as soil pH, microbial activity, and colloid chemistry (Lynch, 2011). Root systems display an array of physiological, morphological, and architectural responses to low P availability. Various root adaptions to low P availability improve the ability of the root to explore the soil, especially the topsoil, where P availability is greatest (Borch *et al.*, 1999; Lynch and Brown, 2001; Ma *et al.*, 2001). Shallower growth angles of axial roots and greater root length density enhance P acquisition in the topsoil (Bonser *et al.*, 1996; Manske *et al.*, 2000; Richardson *et al.*, 2011). Greater production of adventitious roots in dicots enhance topsoil foraging because of their shallow growth angles and reduced metabolic cost (Miller *et al.*, 2003). Root hairs contribute to P acquisition by expanding the physical and chemical exploitation of the rhizosphere (Richardson *et al.*, 2011; Miguel *et al.*, 2015). The formation of root cortical aerenchyma (RCA) increases P acquisition by reducing the metabolic costs of soil exploration (Postma and Lynch, 2011a, b; Galindo-Castañeda *et al.*, 2018), as does root cortical senescence (Schneider *et al.*, 2017a, b).

Lateral roots emerge from axial roots through the formation of lateral root primordia that initiate from pericycle founder cells (Nibau *et al.*, 2008; De Smet, 2012). The formation and growth of lateral roots are among the most important factors governing root system architecture. Lateral roots including any subsequent higher order branches typically comprise the majority of biomass and root length of the root system (Postma *et al.*, 2014). Lateral roots play an important role in P acquisition by increasing soil exploration (Zhu *et al.*, 2005*a*), the absorptive surface of the root system (Xie and Yu, 2003), and P solubilization.

The initiation and elongation of lateral roots is controlled by intrinsic genes. In maize, *rum1*, *Irt1*, *slr1*, and *slr2* affect lateral root elongation and lateral root density in the embryonic primary and seminal roots, but not in crown roots (Hochholdinger and Feix, 1998; Hochholdinger *et al.*, 2001; Von Behrens *et al.*, 2011; Zhang *et al.*, 2014). While the mutants of *aux1* showed the reduction of lateral root number only in the primary root, the mutants of *nal2/3* and *nar2.1* display a reduced number of lateral roots in both the primary and crown root in rice (Cho *et al.*, 2013; Zhao *et al.*, 2015; Huang *et al.*, 2015). In *Arabidopsis thaliana*, auxin response genes ARF7 and ARF19 positively regulate lateral root formation through direct activation of *LBD/ASL* genes (Okushima *et al.*, 2007). *LBD16/ASL18* display an important role in the asymmetrical division of founder cells during lateral root initiation (Goh *et al.*, 2012). Quantitative trait loci (QTLs) associated with natural genetic variation have been identified in several crop species, including maize (Burton *et al.*, 2014).

P deficiency affects lateral root emergence and development in diverse species including common bean (*Phaseolus vulgaris*), maize (*Zea mays*), and *Lupinus albus* (Johnson *et al.*, 1996; Borch *et al.* 1999; Zhu and Lynch, 2004; Chen *et al.*, 2011). In common bean, growth of the main (primary and basal) root axes was maintained under low P availability, while initiation of lateral roots was reduced, so that lateral root density decreased (Borch *et al.* 1999). In maize, some genotypes show an increase in the number and length of lateral roots, while others show the opposite effect under P deficiency (Zhu and Lynch, 2004). In water hyacinth (*Eichhornia crassipes*), P deficiency increases lateral root density and length, while the diameters decrease (Xie and Yu, 2003). Arabidopsis grown in buffered media with suboptimal P availability maintains axial root growth at the expense of lateral rooting (Hanlon 2017). P deficiency enhances the emergence of tertiary lateral roots and induces the formation of proteoid (cluster) roots in white lupin (Johnson *et al.*, 1996), which mobilize rhizosphere P from unavailable pools (Neumann *et al.*, 1999).

**Fig. 1.**
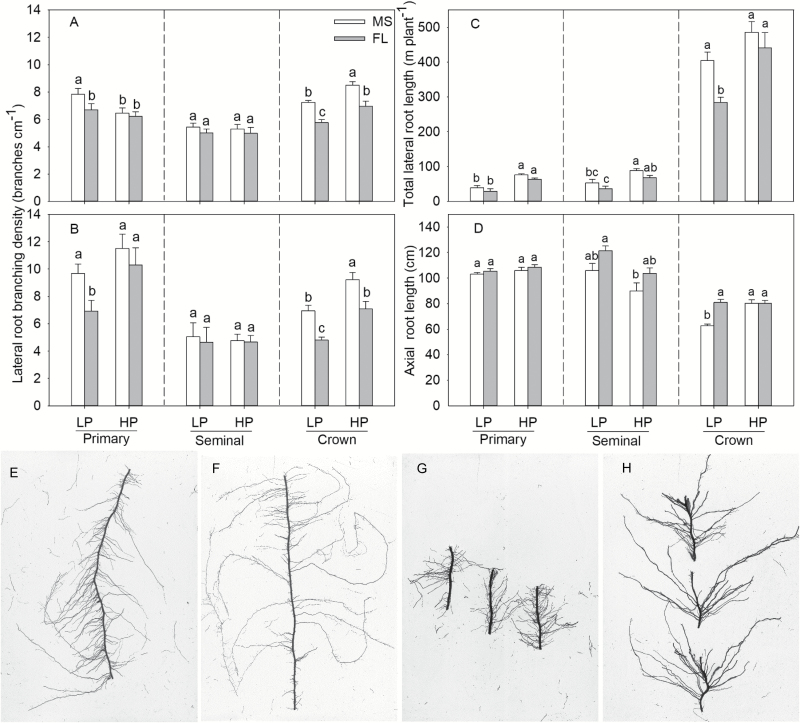
Lateral root branching density of primary, seminal, and crown roots under low phosphorus (LP) and high phosphorus (HP) availability of MS (many short) and FL (few long) lateral root phenotypes at 45 d after planting in greenhouse mesocosms (A), and at the VT stage in the field at Rock Springs (B). Total lateral root length of primary, seminal, and crown roots (C) and axial root length of primary, seminal, and crown roots (D) under low phosphorus (LP) and high phosphorus (HP) availability of MS and FL lateral root phenotypes at 45 d after planting in greenhouse mesocosms; root segment in the greenhouse of MS (E) and FL (F), and in the field of MS (G) and FL (H). The data shown are means of four replicates of the four genotypes in each phenotypic group. Different letters represent significant differences (*P*≤0.05).

Results from the functional–structural model *SimRoot* predicted that the optimal lateral root branching density (LRBD) for soil resource acquisition will be proportional to resource mobility (Postma *et al.*, 2014; Fig. 1). The optimal LRBD for the acquisition of nitrate, water, and sulfate should be low, while the optimal LRBD for the acquisition of phosphate, potassium, ammonium, iron, manganese, copper, and zinc should be high, and the optimal LRBD for the acquisition of calcium and magnesium should be intermediate (Postma *et al.*, 2014). Recent results confirm that root phenotypes with fewer, longer lateral roots are superior to phenotypes with many, short lateral roots under suboptimal availability of nitrogen (N) (Zhan and Lynch, 2015) and water (Zhan *et al.*, 2015), two primary soil resources with high mobility. The prediction that greater LRBD benefits P capture in maize remains unverified by empirical data. In this study, we test the hypothesis that greater LRBD improves P capture from low P soil in maize by increasing root length density in shallow soil horizons.

## Materials and methods

### Plant materials and experiment design

Eight intermated recombinant inbred lines (RILs) of maize (*Zea mays* L.) were obtained from the parental lines B73×Mo17 (Senior *et al.*, 1996; Kaeppler *et al.*, 2000). In previous studies these genotypes had contrasting LRBD and length (Trachsel *et al.*, 2011, 2013; Zhan and Lynch, 2015; Zhan *et al.*, 2015), four RILs with few long (FL) lateral roots and four with many short (MS) lateral roots. All seeds were obtained from Shawn Kaeppler, University of Wisconsin, Madison, WI, USA. This experiment was conducted both in greenhouse mesocosms and in the field in 2014 and 2016.

### Greenhouse mesocosm study

The factors were two P levels: optimum P (HP) and suboptimal phosphorus (LP), and eight genotypes (MS: IBM79, IBM295, IBM301, IBM321; FL: IBM67, IBM86, IBM98, IBM327), in four replicates. A 2 × 8 factorial arrangement of treatments was arranged in a randomized complete block design with position in the greenhouse as the blocking factor.

Mesocosms consisted of polyvinylchloride (PVC) cylinders 15.7 cm in diameter and 155 cm in height, which were lined with transparent hi-density polyethylene plastic sleeves. The growth medium consisted of 29 liters per mesocosm of (volume-based) 50% medium size (0.5–0.3 mm) commercial grade sand (Quikrete Companies Inc., Harrisburg, PA, USA), 35% horticultural size #3 vermiculite, 5% perlite (Whittemore Companies Inc., Lawrence, MA, USA), and 10% topsoil (Typic Hapludalf, pH 6.7, silt loam). KH_2_PO_4_ was mixed into the growth medium (HP, 200 μΜ; LP, 20 μM; 29 liters per mesocosm), and K_2_SO_4_ was added to equalize the K content of LP and HP media once before transplanting. Two days before transplanting, the cylinders were irrigated with 4.7 liters of a nutrient solution adjusted to pH 6.0 and consisting of (in μM): NO_3_ (4500), NH_4_ (300), K (1000), Ca (1750), SO_4_ (1500), Mg (1000), B (46), Mn (9), Zn (7), Cu (0.32), Mo (0.80), and EDTA-Fe (77). Growth conditions consisted of 1200 µmol photons m^−2^ s^−1^ maximum photosynthetically active radiation (PAR), a photoperiod of 14/10 h at 28/24 °C (light/darkness), and 40–70% relative humidity. Seeds of eight genotypes were surface-sterilized in 0.05% NaOCl for 15 min and imbibed for 24 h in aerated 1 mM CaSO_4_, then were placed in darkness at 28 ± 1 °C for 2 d. Three seedlings of similar size were transplanted to mesocosms, and they were thinned to one plant after 7 d. A 200 ml aliquot of nutrient solution was applied to each mesocosm every 2 d via drip irrigation with a DI-16 Dosatron fertilizer injector (Dosatron International Inc., Dallas, TX, USA).

#### Chlorophyll content and leaf photosynthesis

Plants were harvested 45 d after transplanting. Two days before harvest, leaf gas exchange of the youngest fully expanded leaf was measured with a Licor-6400 Infrared Gas Analyzer (Li-COR Biosciences, Lincoln, NE, USA) using red–blue light at 1200 μmol photons m^−2^ s^−1^, constant CO_2_ concentration of 400 ppm, and leaf temperature of 25 °C. At harvest, the youngest fully expanded leaf was sampled 20 times with a hole punch, then stored at –20 °C for determination of leaf chlorophyll content. Leaf chlorophyll was extracted with 95% ethanol in water and calculated by Arnon’s (1949) equations.

#### Root respiration

Root respiration of axial and lateral roots was measured. Three 10 cm root segments from the second whorl of crown roots were excised 8 cm from the base. Lateral roots of axial roots were removed with a Teflon blade (Electron Microscopy Sciences, Hatfield, PA, USA). Excised axial and lateral root samples were patted dry and placed in a 40 ml custom chamber connected to the Li-6400 IRGA (LI-COR) separately. The temperature of the chamber was maintained at 25 ± 1 °C using a water bath while respiration was measured. Carbon dioxide evolution from the root segments was recorded every 5 s for 180 s. After root respiration measurement, segments of axial and lateral roots were sampled for dry weight determination.

#### Anatomical analysis

Three 2 cm root segments from the second whorl of crown roots were excised 20 cm from the base and stored in 95% alcohol before analysis. Root segments were ablated using laser ablation tomography to obtain images for anatomical analysis. Briefly, laser ablation tomography is a semi-automated system that uses a laser beam (Avia 7000; 355 nm pulsed laser) to vaporize or sublimate the root at the camera focal plane ahead of an imaging stage. The sample is incremented, vaporized, or sublimated, and imaged simultaneously. Cross-sectional images were taken using a Canon T3i camera with a 53 micro lens (MP-E 65 mm) on the laser-illuminated surface. Root images were analyzed using RootScan, an image analysis tool developed for analyzing root anatomy (Burton *et al.*, 2012).

#### Shoot and root sampling and analysis

The shoot was cut at the growth medium surface, and then determined for biomass after drying at 60 °C for 72 h. Roots were separated from the soil by vigorously rinsing at low pressure with water. Roots were extracted from each 20 cm soil depth increment; three 5 cm root segments were taken from each whorl of crown root, primary root, and seminal root (8 cm from the top of every soil depth). Subsequently extracted root samples were scanned (Epson, Perfection V700 Photo) at a resolution of 23.6 pixels mm^–1^ (600 dpi) and analyzed using WinRhizo Pro (Régent Instruments, Québec, Canada) to obtain lateral root length, lateral root number, and total root length. Three roots were randomly chosen from each whorl of crown roots and seminal roots to determine axial root length.

### Field study

#### Experimental site

Experiments were conducted during May to September in 2014 and 2016 at the Russell E. Larson Agricultural Research Center of the Pennsylvania State University in Rock Spring, PA, USA (RS) (40°42′37″52 N, 77°57′07″54 W, 366 masl). The soil at the experimental sites is a Hagerstown silt loam (fine, mixed, semiactive, mesic Typic Hapludalf).

#### Plant materials, experimental design, and field conditions

Over many years, plots with contrasting P availability were created by differential application of P fertilizer, with all other management treatments held constant. The P content of the soil was 37.5 ± 1.8 mg kg^−1^ for the HP treatment and 10.2 ± 0.5 mg kg^−1^ for the LP treatment. Other nutrients were adjusted to meet the requirements for maize production as determined by soil testing. Pest control and irrigation were carried out as needed. Seeds of eight IBM RILs (the same as those used in the mesocosm study) were planted on 4 June 2014 and 26 May 2016. The experiments were arranged in a randomized complete block design replicated four times, with each block consisting of alternating HP and LP domains in which genotypes were randomly arranged. Individual plots consisted of three rows each 4.2 m long, 75 cm wide, and distance within a row was 23 cm, resulting in a planting density of 6 plants m^−2^.

#### Sampling and analysis

Shoots and roots were harvested at the VT stage (10 weeks after planting). Two days before harvest, the net photosynthesis rate (*P*n) was measured on the ear leaf at 1800 μmol photons m^–2^ s^–1^ PAR, constant CO_2_ concentration of 400 ppm, and leaf temperature of 25 °C using a Li-Cor 6400. At harvest, the ear leaf was sampled at 20 locations with a hole punch, then stored at –20 °C for determination of leaf chlorophyll content.

Two adjacent plants per replicate were randomly selected in the same row for shoot dry weight and dried at 60 °C for 72 h before being weighed. Ground tissue was ashed at 500 °C and analyzed for P content spectrophotometrically (Murphy and Riley, 1962).

Roots were excavated in a soil volume 40 cm in diameter and 25 cm in depth from the plant stem and then cleaned followed the shovelomics method (Trachsel *et al.*, 2011). The clean roots were subsequently used to measure lateral root number. Three 5 cm root segments were taken 3 cm from the base of each whorl of the crown root, primary root, and seminal root, and lateral root number of the corresponding roots was based on counts. All roots emerging below ground were classified as crown roots.

Soil cores were taken (5.1 cm×60 cm, Giddings Machine Co., Windsor, CO, USA), midway between two plants within a row. Cores were subdivided into 10 cm segments and washed by low pressure water. Washed roots were scanned and analyzed as described above to obtain root length at each soil depth. Percentages of root length at each depth were calculated in each soil core. Depth above which 95% (D_95_) of root length is located was calculated by linear interpolation between the cumulative root lengths as described in Trachsel *et al.* (2013).

### Data analysis

The experimental data were statistically analyzed by ANOVA with SPSS (SPSS Inc. Release 2008. Version 17.0. SPSS Inc., Chicago). Two-way ANOVAs were used for comparisons between FL and MS lines, P levels, and the interaction between these main effects. Tukey’s Honest Significant Difference method (α=0.05) was used for multiple comparisons. Linear regression analysis and Pearson correlation coefficients were calculated by Sigmaplot (Sigmaplot 12.5, Systat Software Inc., CA, USA).

## Results

### Lateral root branching and length

MS lines had greater LRBD of crown roots than FL lines, with no significant difference in primary and seminal roots in mesocosms (Fig. 1A; Supplementary Fig. S1A at *JXB* online). P deficiency significantly decreased the LRBD of crown roots, but did not influence the LRBD of primary and seminal roots in either group. In the field, MS lines had more LRBD of crown roots than FL lines, and also had greater LRBD of primary roots than FL lines under LP conditions (Fig. 1B; Supplementary Fig. S2). In the field, low P availability decreased the LRBD of crown roots of both groups and the primary roots of FL lines, but did not affect the LRBD of seminal roots.

**Fig. 2.**
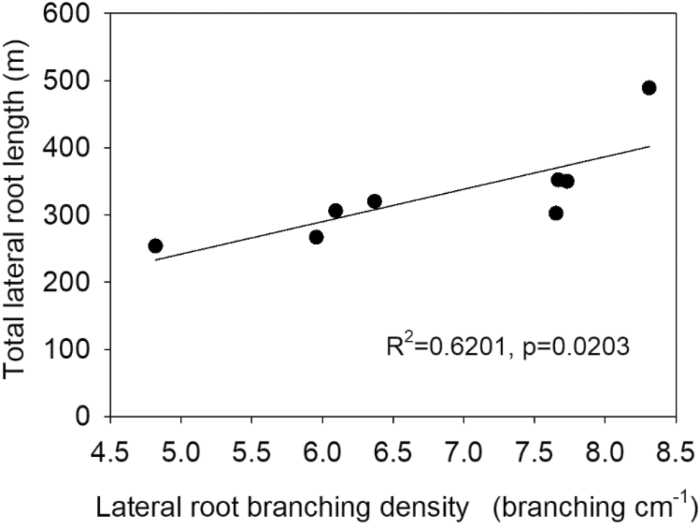
Correlation of total root length with lateral root branching density of crown roots in the greenhouse under low phosphorus. Each point is the mean of four replicates of each genotype.

In mesocosms, both phenotypes had equivalent total lateral root length in HP conditions (Fig. 1C). Low P availability reduced the lateral root length of primary, seminal, and crown roots, except for crown roots of MS lines, which were not affected by P availability (Fig. 1C). Total lateral root length was correlated with LRBD of crown roots under LP conditions (Fig. 2). Low P availability reduced axial root length (ARL) of crown roots in MS lines but not FL lines (Fig. 1D). ARL did not differ between groups under high P.

### Root depth

**Fig. 3.**
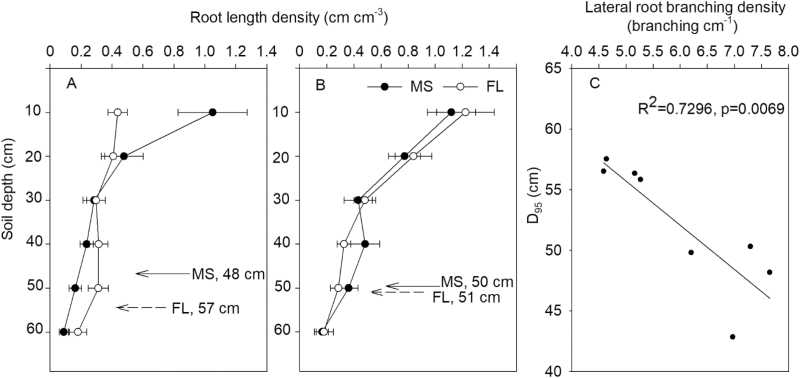
Root length density and D_95_ of maize lines with MS (many short) and FL (few long) lateral root phenotypes at the VT stage in the field under low phosphorus (A) and high phosphorus (B) availability. Correlation of D_95_ with lateral root branching density of crown roots in the field under low phosphorus availability (C). Data shown are means of four replicates of the four genotypes in each phenotypic class in either HP or LP ±SE.

In the field, low P availability decreased root length density, and MS lines had greater root length density in 0–20 cm soil depth under LP conditions (Fig. 3A, B). MS lines had shallower rooting depth (D_95_) than FL lines under LP conditions, and D_95_ was negatively correlated with LRBD of crown roots (Fig. 3C).

### Phosphorus uptake and uptake efficiency

**Fig. 4.**
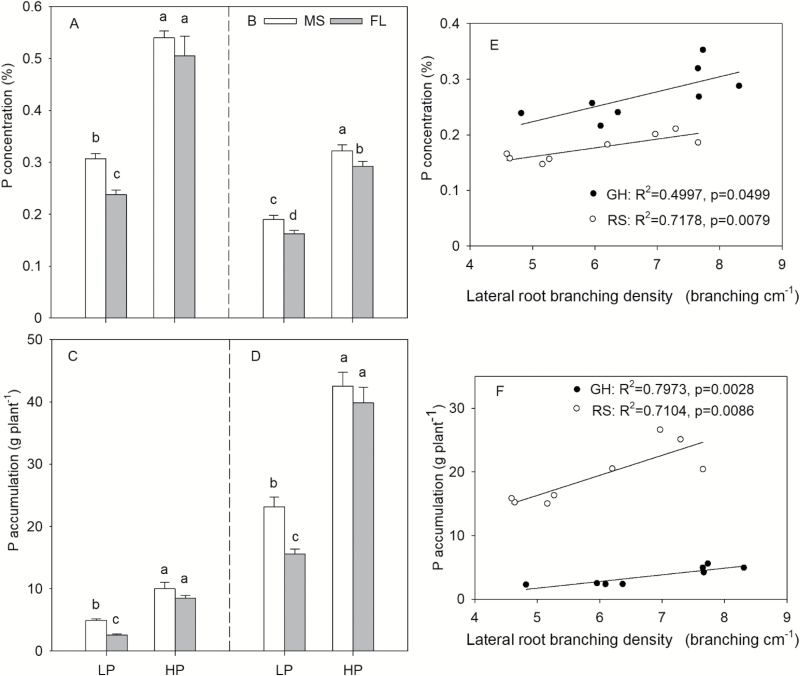
Phosphorus concentration and accumulation of maize lines with MS (many short) and FL (few long) lateral root phenotypes under high and low phosphorus availability in greenhouse mesocosms (A, C) and at the VT stage in the field (B, D). Correlation of P concentration (E) and P accumulation (F) with lateral root branching density of crown roots in mesocosms (GH) and the field (RS) under low phosphorus availability (LP). Data shown are means of four replicates of the four genotypes in each phenotypic class in either HP or LP ±SE. Different letters represent significant differences (*P*≤0.05).

In mesocosms, low P availability significantly decreased the P concentration of MS lines and FL lines by 43% and 53%, respectively (Fig. 4A). In LP, MS lines had a 29% greater P concentration than FL lines (Fig. 4A). Low P availability reduced P accumulation in mesocosms by 51% in MS lines and 70% in FL lines (Fig. 4C). In LP, MS lines had 89% greater P accumulation than FL lines (Fig. 4C). In the field, low P availability significantly decreased the P concentration of MS lines and FL lines by 41% and 44%, respectively (Fig. 4B). MS lines increased P concentration by 17% and 11%, respectively, in LP and HP conditions compared with FL lines (Fig. 4B). Low P availability reduced P accumulation in the field by 46% in MS lines and by 61% in FL lines (Fig. 4D). In LP, MS lines had 49% greater P accumulation compared with FL lines (Fig. 4D). P concentration and accumulation were positively correlated with lateral branching density of crown roots under low P in both the greenhouse and the field (Fig. 4E, F).

### Root and shoot growth

**Fig. 5.**
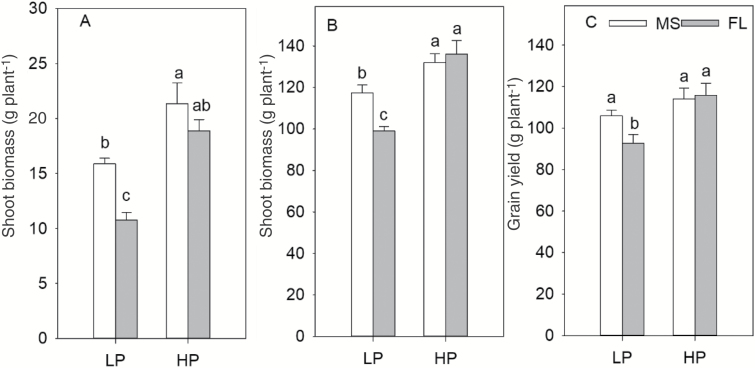
Shoot biomass of maize lines with MS (many short) and FL (few long) lateral root phenotypes under high phosphorus (HP) and low phosphorus (LP) availability at 45 d after planting in greenhouse mesocosms (A), shoot biomass (B), and grain yield (C) under high phosphorus and low phosphorus conditions in the field at the VT stage. The data shown are means of four replicates of the four genotypes in each phenotype in either HP or LP ±SE. Different letters represent significant differences (*P*≤0.05).

**Fig. 6.**
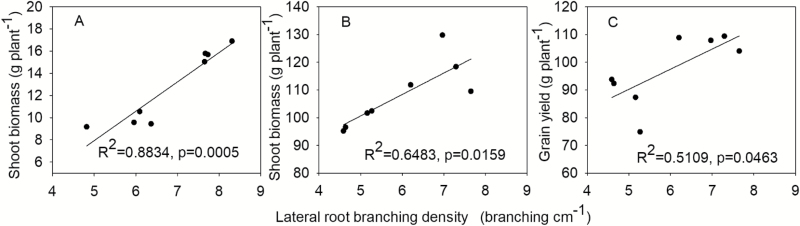
Correlation of shoot biomass under low phosphorus (LP) availability at 45 d after planting in greenhouse mesocosms (A), shoot biomass (B), and grain yield (C) under low phosphorus conditions in the field in Rock Spring (RS) at the VT stage with lateral root branching density of crown roots. Each point is the mean of four replicates of each genotype.

In mesocosms, LP significantly decreased shoot biomass of both FL and MS lines (Fig. 5A; Table 1). Shoot biomass showed no significant difference under HP (Fig. 5A). Under LP, MS lines had 48% greater shoot biomass than FL lines (Fig. 5A). In the field, low P availability reduced the shoot biomass of MS and FL lines by 11% and 27%, respectively (Fig. 5B). Under LP, MS lines had 19% greater shoot biomass than FL lines (Fig. 5B). LP reduced grain yield in MS and FL lines by 10% and 18%, respectively (Fig. 5C). Under LP, MS lines had 14% greater yield than FL lines (Fig. 5C). Shoot biomass and grain yield were positively correlated with lateral branching density of crown roots under LP both in mesocosms and in the field (Fig. 6A–C).

**Table 1.** Summary of ANOVA for physiological parameters as influenced by P and phenotype (PT) in the greenhouse mesocosm experiment (GH) and in the field experiment at Rock Springs (RS)

	Effect	LRBD	Pn	CHLC	SPC	PUA	PB	GY
GH	P	74.07**	6.26*	4.11*	133.9***	78.37**	16.72**	
	PT	49.57**	5.33*	5.32*	5.90*	9.41*	7.14*	
	P×PT	5.28*	4.99*	1.49^†^	0.76^†^	0.39^†^	3.66*	
RS	P	118.4***	18.04**	5.52*	268.7***	172.6***	27.2**	11.68**
	PT	121.2***	7.52*	7.11*	30.12**	4.04*	4.07*	2.72^†^
	P×PT	5.54*	5.15*	1.99^†^	4.28*	5.05*	8.33*	4.19**

The associated *F*-values and probabilities (^†^*P*≤0.1, *P≤0.05, **P≤0.001, ***P≤0.0001) are shown. Lateral root branching density of crown roots (LRBD), plant biomass (PB), photosynthesis rate (*P*n), chlorophyll content (CHLC), shoot phosphorus content (SPC), phosphorus uptake amount (PUA), and grain yield (GY).

**Fig. 7.**
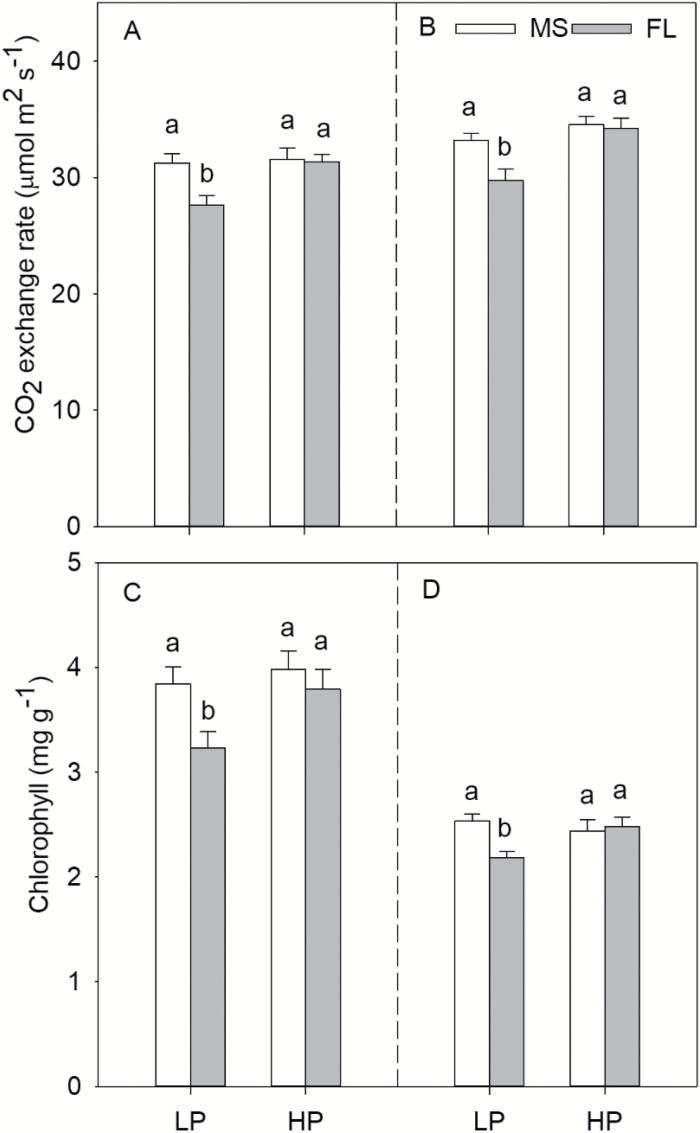
Leaf CO_2_ exchange rate and chlorophyII content of maize lines with MS (many short) and FL (few long) lateral root phenotypes under high phosphorus (HP) and low phosphorus (LP) availability at 45 d after planting in greenhouse mesocosms (A, C) and at the VT stage in the field (B, D). The data shown are means of four replicates of the four genotypes in each phenotype in either HP or LP ±SE. Different letters represent significant differences (*P*≤0.05).

In mesocosms and the field, leaf photosynthetic rates showed no difference in MS and FL lines under HP. LP had no effect on the leaf photosynthetic rate of MS lines, but reduced that of FL lines by 9% and 13%, respectively, in mesocosms and in the field (Fig. 7A, B). Under LP conditions in mesocosms, MS lines had a greater leaf photosynthesis rate than FL lines (Fig. 7A). In the field, the leaf photosynthetic rate of MS was 12% greater than that of FL lines under LP (Fig. 7B). In mesocosms and in the field, the chlorophyll content had the same trend as the leaf photosynthetic rate (Fig. 7C, D).

Benefits of the MS phenotype under P stress for root growth, root respiration, P uptake, photosynthesis, plant biomass and yield in 2014 were similar to those in 2016 (Supplementary Figs S1–S8).

## Discussion

Our results support the hypothesis that in soil with low P availability, the phenotype of many, short lateral roots (‘MS’) is superior to fewer, longer lateral roots (‘FL’), as evidenced by shallower rooting depth, greater P acquisition, and therefore greater photosynthesis, leaf chlorophyll content, plant biomass, and yield (Figs 1C, 2–7). Under low P availability in mesocosms, MS lines had greater total lateral root length, photosynthesis, P accumulation, and shoot biomass than FL lines (Figs 1C, 2, 4–7). Under LP in the field, MS lines had shallower rooting depth, greater P accumulation, and greater photosynthesis, shoot biomass, and yield than FL lines (Figs 3–7).

The fact that we observed comparable benefits of the MS phenotype in two field seasons and in greenhouse mesocosms, and that these empirical results validate *in silico* predictions (Postma *et al.*, 2014), reduces the probability that our results are confounded by biophysical or biotic factors present in any specific environment. For example, mycorrhizal symbiosis is important for P mobilization and capture, but whereas this symbiosis was present in the field studies it was absent in the ‘SimRoot’ modeling and the mesocosm study, so the consistency of our results across environments argues against a role for mycorrhizal symbiosis (and other microbial interactions) in mediating the benefits of the MS phenotype.

RILs with contrasting lateral root phenotypes were used for the analysis of the physiological function of lateral root branching. RILs share a common genetic background, descending from the same parental lines B73×Mo17, but each RIL is a distinct genotype. Lateral root formation in maize is governed by multiple genetic loci, thus RILs are an appropriate means to compare the physiology of related genotypes that contrast for lateral rooting, since the genetic control of this phenotype is poorly understood and complex (Zhu and Lynch, 2004). The development of near isogenic lines (NILs) contrasting only in one or several genes would not be possible given the current state of knowledge. Many such NILs would be needed as different sets of genes would have to be contrasted. Single gene variants are useful for studies of gene function, but are not suited to phenotypic traits controlled by many genes (Hochholdinger *et al.*, 2001; Zhang *et al.*, 2014). The focus of this study is, however, not gene function but phene function, i.e. the functional utility of a given phenotype. RILs are ideally suited for this purpose since they contrast for the phene of interest yet share a common genetic background (i.e. they descend from the same two parents), which minimizes the likelihood that results are confounded by extraneous genetic effects.

We observed large phenotypic variation in LRBD in maize (1–41 cm^−1^ major axis), suggesting that LRBD has varying utility and trade-offs in specific environments (Lynch, 2013). Both the nutrient and carbon status of the plant and the local nutrient environment of the root tip influence LRBD. The growth rate of lateral roots was more sensitive to carbon availability than the growth of axial roots, and there is a trade-off between the number of laterals and the average length of laterals (Postma *et al.*, 2014). Maize genotypes differ in their lateral branching responses to low P availability, with some genotypes increasing and others decreasing branch density and length (Zhu and Lynch, 2004; Zhu *et al.*, 2005*a*). In the present study, phenotypes had significant and stable differences in LRBD, with MS lines having greater lateral root number and length than FL lines (Fig. 1A, B; Supplementary Figs S1, S2). In a heterogeneous soil environment, complex architecture and plasticity are controlled by multiple genes to enhance soil resource acquisition. Genetic dissection of root formation in maize indicates that different root classes had distinct LRBD phenotypes because they are under distinct genetic control (Hochholdinger *et al.*, 2004). In the present study, lateral root branching density differed among crown roots, primary roots, and seminal roots (Fig. 1A, B; Supplementary Figs S1, S2).

Reduced LRBD is valuable for water and nitrogen acquisition (Zhan and Lynch, 2015; Zhan *et al.*, 2015). Reduced LRBD reduces the metabolic costs of soil exploration, permitting greater axial root elongation, greater rooting depth, and thereby greater water and nitrogen capture under water and nitrogen stress conditions (Zhan and Lynch, 2015; Zhan *et al.*, 2015). These results support the SCD (steep, cheap, and deep) ideotype that few but long axial root laterals contribute to deeper soil exploration under limiting N or water (Lynch, 2013). In the present study, lateral branching of crown roots had substantial effects on plant P acquisition under low P availability. MS lines had greater P uptake than FL lines in LP soil (Fig. 4A, B). We interpret the contrasting utility of LRBD for water, N, and P capture as a function of resource mobility. Water and N (in the form of nitrate, which is the predominant form of available N in most agricultural soils) are mobile, and in typical maize production environments leach downwards in the soil profile over time (Lynch and Wojciechowski, 2015). In contrast, P is highly immobile and its availability is greatest in shallow soil strata (Lynch, 2011). While reduced LRBD permits greater axial elongation and therefore deeper rooting and greater capture of water and N, increased LRBD diverts resources to lateral root growth over axial elongation, which promotes more intensive exploration of shallow soil strata, thereby improving P capture.

Optimal LRBD within a root system is proposed to co-optimize topsoil foraging for P and subsoil foraging for water and N by greater LRBD in the topsoil and reduced LRBD with depth (Postma *et al.*, 2014). Our results are consistent with that proposal. The substantial genotypic variation for LRBD in maize and other species, combined with the importance of such variation for the acquisition of the three primary soil resources of water, N, and P, suggest that optimized LRBD merits attention as a breeding target for crop improvement.

## Supplementary data

Supplementary data are available at *JXB* online.

**Table S1.** Summary of ANOVA for lateral root branching density of crown roots, plant biomass, axial root respiration rate, lateral root respiration rate, photosynthetic rate, chlorophyll content, phosphorus uptake amount, phosphorus uptake efficiency, grain yield as influenced by phosphorus and phenotype in the greenhouse mesocosm experiment and in the field experiment at Rock Spring

**Fig. S1.** Lateral root branching density and total lateral root length of crown, primary, and seminal roots under high P (HP) and low P (LP) availability of MS (many short) and FL (few long) lateral root phenotypes at 45 d after planting in greenhouse mesocosms.

**Fig. S2.** Lateral root branching density of primary, seminal, and crown roots of maize lines with ‘many short’ (MS) or ‘few long’ (FL) lateral root phenotypes under high phosphorus (HP) and low phosphorus (LP) availability at the VT stage in the field.

**Fig. S3.** Shoot biomass and root biomass of maize lines with ‘many-short’ (MS) or ‘few-long’ (FL) lateral root phenotypes under high phosphorus (HP) and low phosphorus (LP) availability at 45 d after planting in greenhouse mesocosms (GH).

**Fig. S4.** Shoot biomass and grain yield of maize lines with ‘many short’ (MS) or ‘few long’ (FL) lateral root phenotypes under high phosphorus (HP) and low phosphorus (LP) availability in the field at the VT stage.

**Fig. S5.** Correlation of plant biomass with lateral root branching density of maize crown roots under high phosphorus (HP) and low phosphorus (LP) availability at 45 d after planting in greenhouse mesocosms and the VT stage in the field.

**Fig. S6.** Leaf photosynthetic rate and SPAD value of maize lines with ‘many short’ (MS) or ‘few long’ (FL) lateral root phenotypes under high phosphorus (HP) and low phosphorus (LP) availability at 45 d after planting in greenhouse mesocosms (A, C), and at the VT stage in the field (B, D).

**Fig. S7.** Phosphorus concentration, accumulation, and uptake efficiency of maize lines with ‘many short’ (MS) or ‘few long’ (FL) lateral root phenotypes under high phosphorus (HP) and low phosphorus (LP) availability at the VT stage in the field.

**Fig. S8.** Correlation of phosphorus accumulation and uptake efficiencywith lateral root branching density of crown roots of maize lines with ‘many short’ (MS) or ‘few long’ (FL) lateral root phenotypes at the VT stage in the field under high phosphorus (HP) and low phosphorus (LP) availability.

**Fig. S9.** Correlation of root surface area with lateral root branching density of crown roots in the greenhouse under low phosphorus (LP).

**Fig. S10.** Principal components analysis conducted with root and shoot variables in mesocosms under low P availability (LP).

**Fig. S11.** Principal components analysis conducted with root and shoot variables in the field under low P availability (LP).

**Fig. S12.** Meteorological data in 2014 and 2016 in Rock Spring: daily average temperature, precipitation, and radiation.

**Fig. S13.** Root hair length and root hair density of MS (many short) and FL (few long) lateral root phenotypes at the seedling stage.

## Supplementary Material

Supplementary Table and Figures
