# Supplementary material for: Greater lateral root branching density in maize improves phosphorus acquisition from low phosphorus soil
Source: J Exp Bot. 2018 Jul 26;69(20):4961–70. doi: 10.1093/jxb/ery252 (PMC6137997; doi:10.1093/jxb/ery252)
Supplement: Supplementary Table and Figures [file ery252_suppl_supplementary_table_s1_figures_s1_s13.pdf]

Supplemental Table S1. Summary of analysis of variance for lateral root branching density of crown root (LRBD), plant biomass (PB), photosynthesis rate (Pn), chlorophyll content (SPAD), phosphorus uptake amount (PUA), phosphorus uptake efficiency (PUE), grain yield (GY) as influenced by phosphorus (P) and phenotype (PT) in the greenhouse mesocosm experiment (GH) and in the field experiment at Rock Springs (RS). The associated F-values and probabilities ($^{\dagger}p\leq0.1$, $^{*}p\leq0.05$, $^{**}p\leq0.001$, $^{***}p\leq0.0001$) are shown.

	Effect	LRBD	PB	Pn	SPAD	PUA	PUE	GY
GH	**Block**	0.73ns	7.79*	0.49ns	0.01*			
	**P**	43.08**	343.05***	99.35***	0.00***			
	**PT**	150.62***	25.96**	24.91**	0.17 ns			
	**P * PT**	5.28*	14.91*	7.05*	0.00*			
RS	**Block**	8.10*	4.69*	0.05ns	3.35†	7.79†	1.07ns	9.35*
	**P**	194.78***	45.23**	30.18*	24.36 *	400.77***	333.49***	101.76***
	**PT**	783.72***	3.68*	0.06ns	1.24ns	10.02*	141.80***	7.9748*
	**P * PT**	5.54*	5.47*	6.71*	5.73*	18.60*	7.09*	20.64*

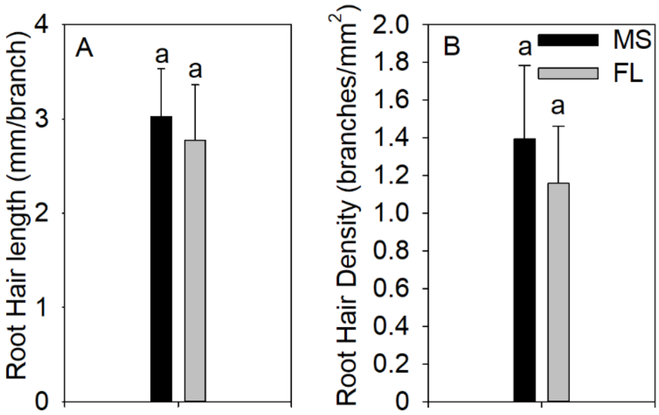

Supplemental Fig. S13 Root hair length (A) and root hair density (B) of MS (many-short) and FL (few-long) lateral root phenotypes at seeding stage.

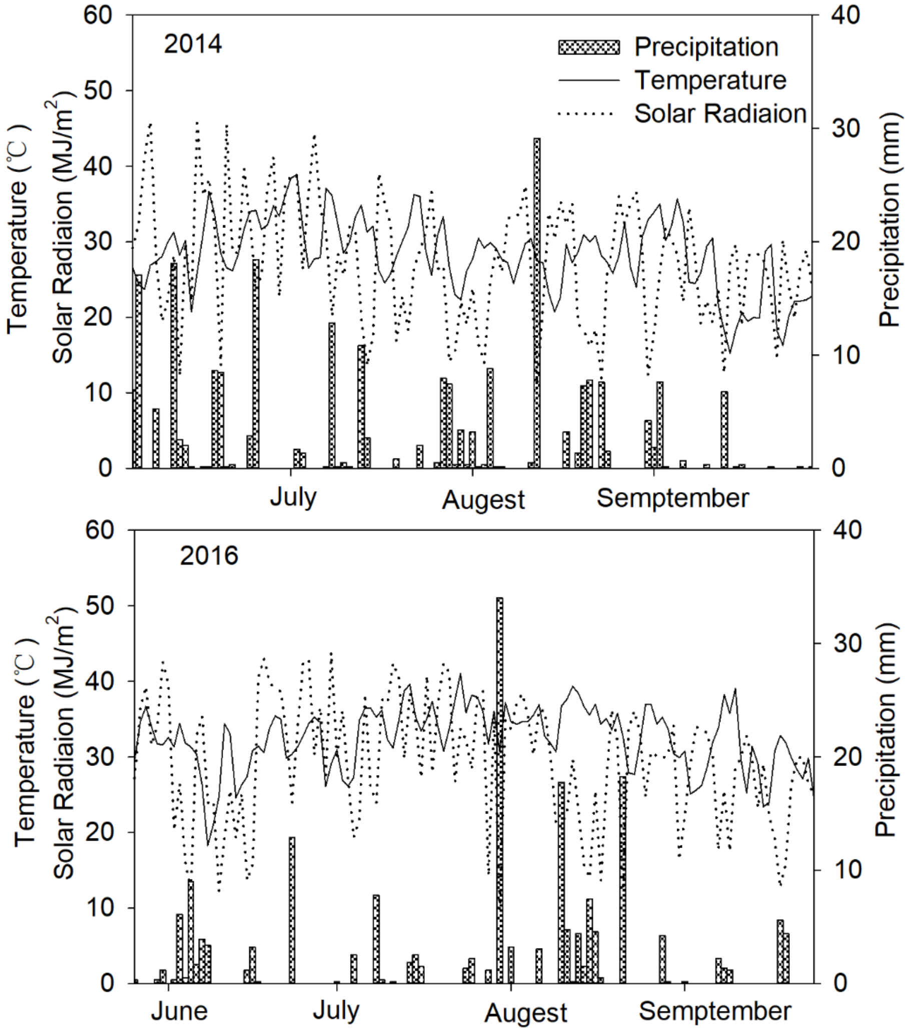

Supplemental Fig. S12 Meteorological data in 2014 and 2016 in Rock Spring of daily average temperature (°C), precipitation (mm) and radiation (MJ/m^2).

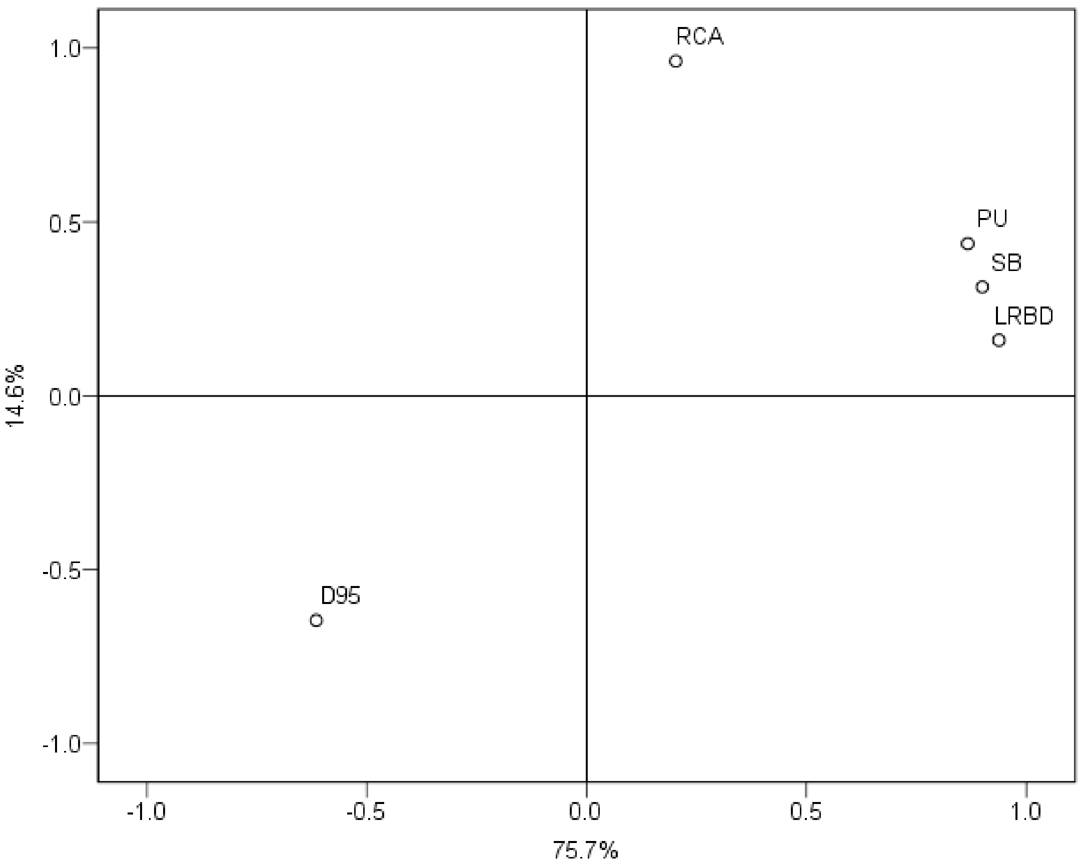

Supplemental Fig. S11 Principal components analysis conducted with root and shoot variables in field under low P (LP) availability. LRBD: lateral root branching density; PU: phosphorus uptake; SB: shoot biomass; RCA: root cortical aerenchyma

.

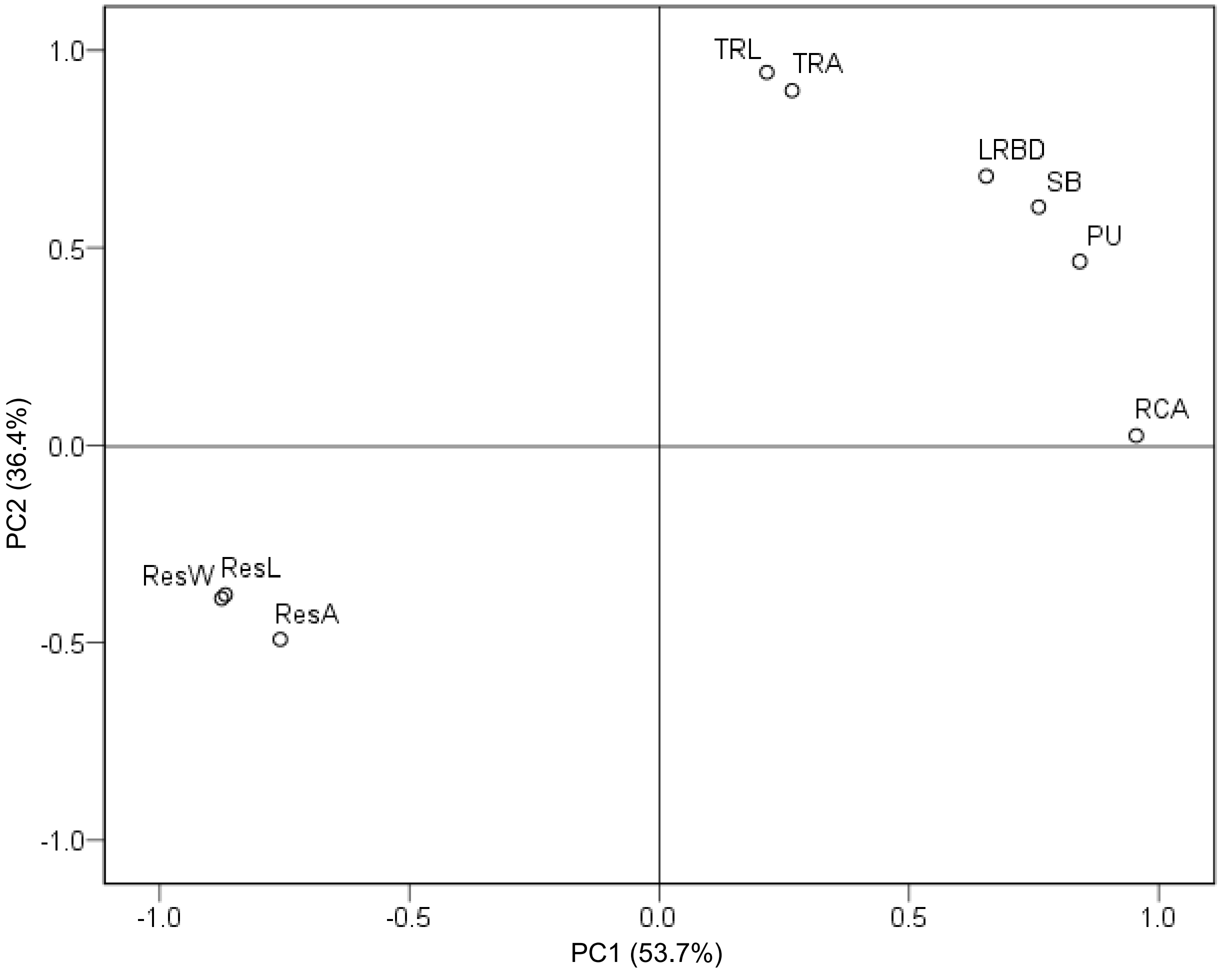

Supplemental Fig. S10 Principal components analysis conducted with root and shoot variables in greenhouse under low P (LP) availability. LRBD: lateral root branching density; TRL: total root length; TRA: total root surface area; PU: phosphorus uptake; SB: shoot biomass; RCA: root cortical aerenchyma; ResW: root respiration of whole root; ResL: root respiration of lateral root; ResA: root respiration of axial root.

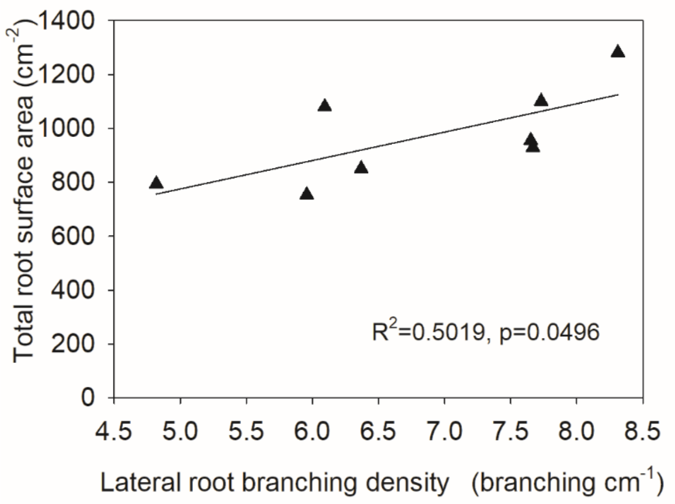

Supplemental Fig. S9 Correlation of root surface area with lateral root branching density of crown roots in the greenhouse under low phosphorus (LP). Each point is the mean of 4 replicates of each genotype.

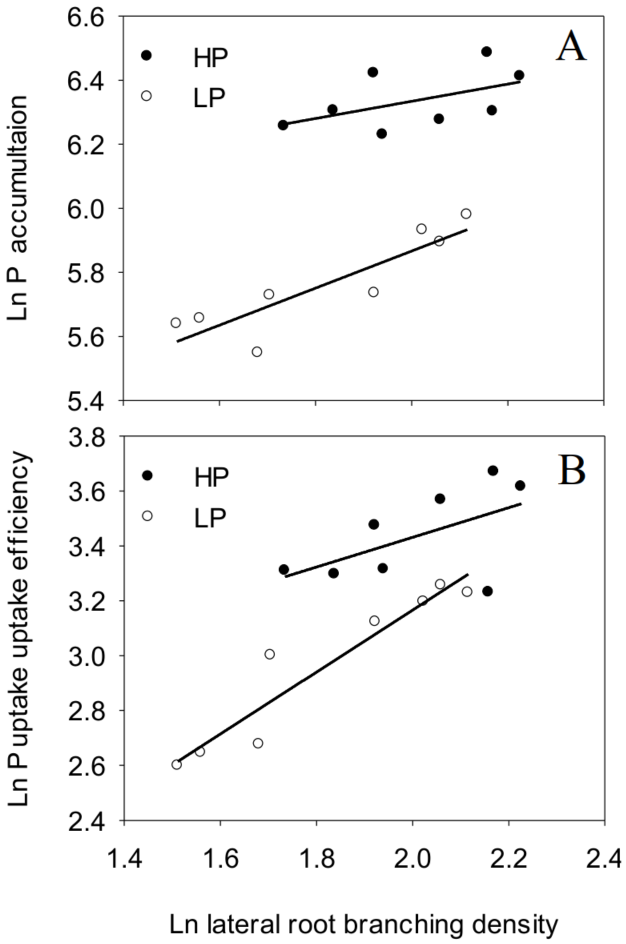

Supplemental Fig. S8 Correlation of phosphorus accumulation (A, HP: R^2=0.2627, p<0.0001; LP: R^2=0.7832, p<0.0001) and uptake efficiency (B, HP: R^2=0.3171, p=0.0113; LP: R^2=0.9.41, p=0.0167) with lateral root branching density of crown roots of maize lines with 'many-short' (MS) or 'few-long' (FL) lateral root phenotypes at VT stage in the field under high P (HP) and low P (LP) availability. Each point is the mean of 4 replicates of each genotype.

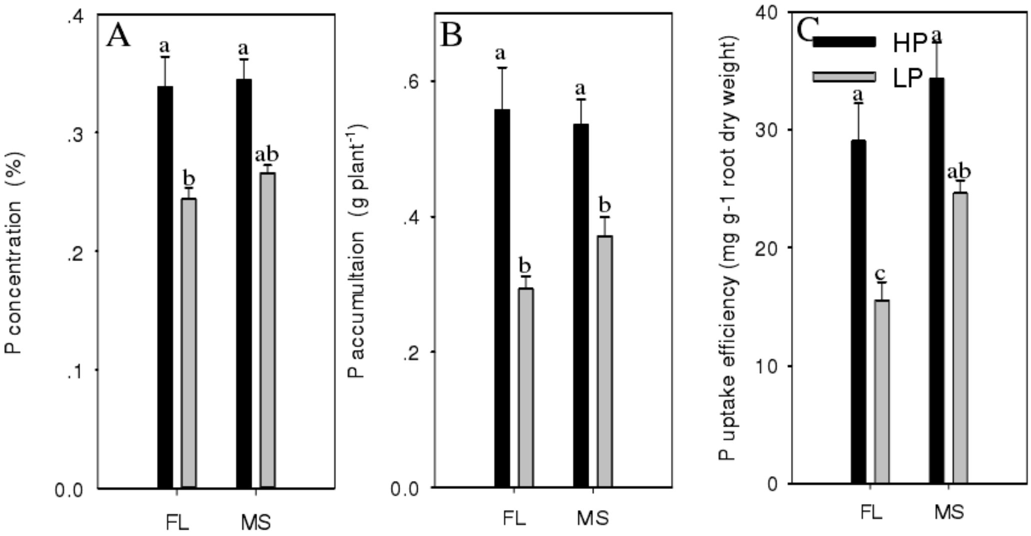

Supplemental Fig. S7 Phosphorus concentration, accumulation and uptake efficiency of maize lines with ‘many-short’ (MS) or ‘few-long’ (FL) lateral root phenotypes under high phosphorus (HP) and low phosphorus (LP) availability at VT stage in the field. The data shown are means of 4 replicates of the 4 genotypes in each phenotypic group in either HP or LP ± SE. Different letters represent significant differences ($p \leq 0.05$).

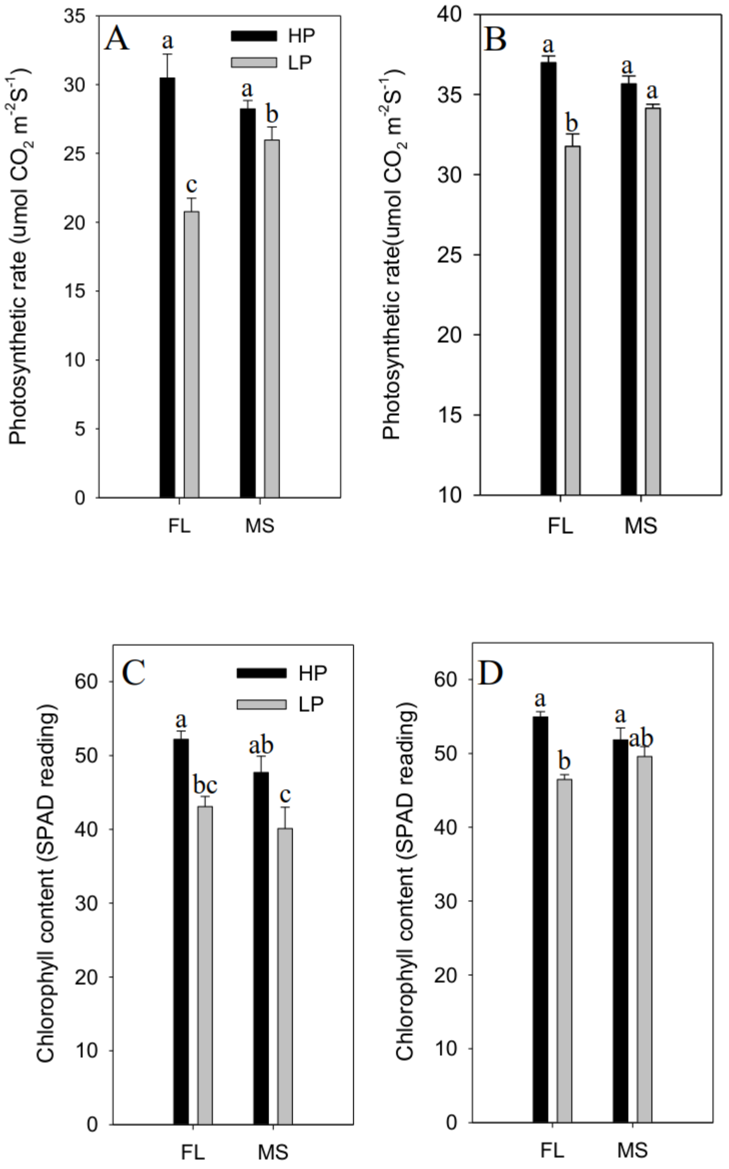

Supplemental Fig. S6 Leaf photosynthetic rate and SPAD value of maize lines with 'many-short' (MS) or 'few-long' (FL) lateral root phenotypes under high phosphorus (HP) and low phosphorus (LP) availability at 45 days after planting in greenhouse mesocosms (A, C), and at VT stage in the field (B, D). The data shown are means of 4 replicates of the 4 genotypes in each phenotypic group in either HP or LP ± SE. Different letters represent significant differences ($p \le 0.05$).

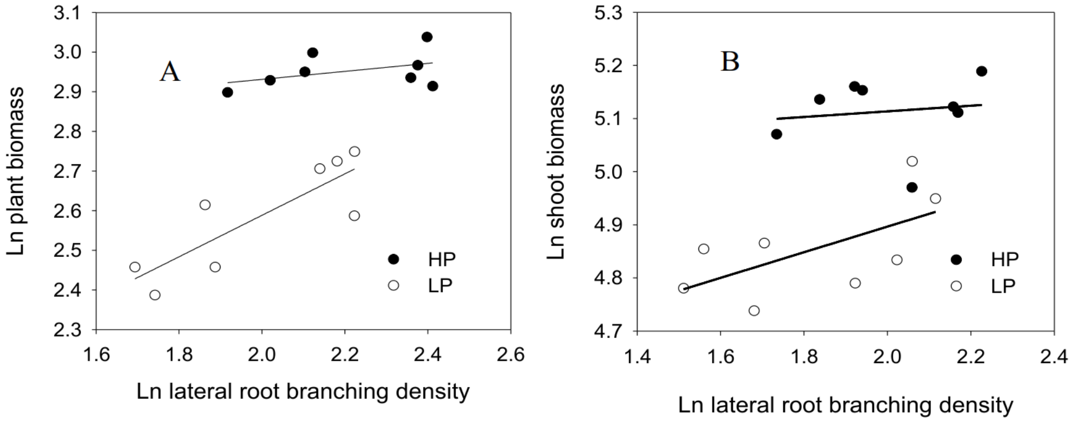

Supplemental Fig. S5 Correlation of plant biomass with lateral root branching density of maize crown roots under high phosphorus (HP) and low phosphorus (LP) availability at 45 days after planting in greenhouse mesocosms (A, HP: R^2=0.1711, p<0.0001; LP: R^2=0.7045, p=0.0014) and VT stage in the field (B, HP: R^2=0.0186, p<0.0001; LP: R^2=0.3807, p<0.0001). Each point is the mean of 4 replicates of each genotype.

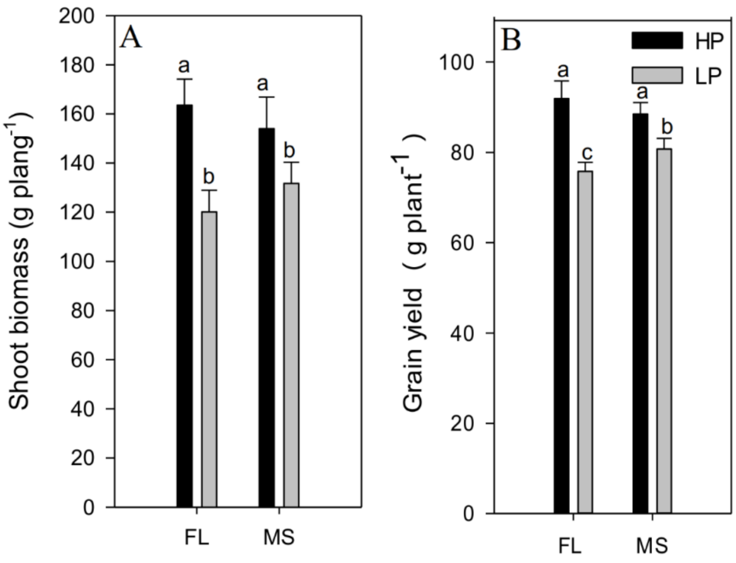

Supplemental Fig. S4 Shoot biomass (A) and grain yield (B) of maize lines with ‘many-short’ (MS) or ‘few-long’ (FL) lateral root phenotypes under high phosphorus (HP) and low phosphorus (LP) availability in the field at VT stage. The data shown are means of 4 replicates of the 4 genotypes in each phenotypic class in either HP or LP ± SE. Different letters represent significant differences ($p \leq 0.05$).

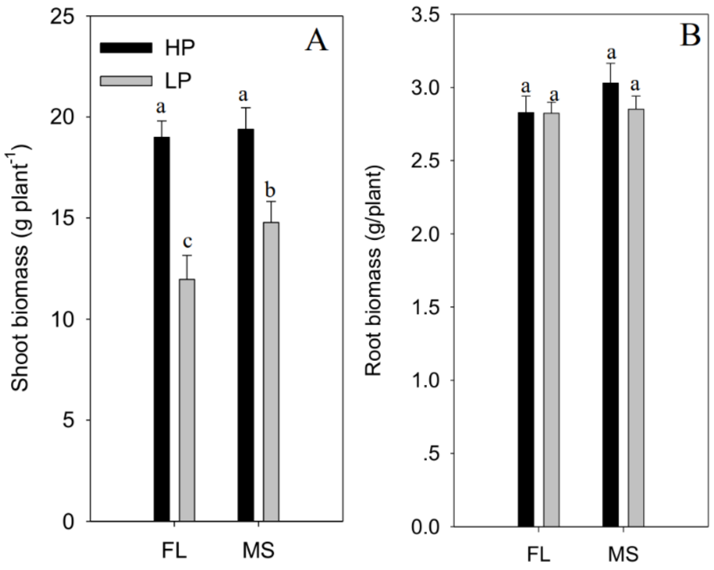

Supplemental Fig. S3 Shoot biomass (A) and root biomass (B) of maize lines with ‘many-short’ (MS) or ‘few-long’ (FL) lateral root phenotypes under high phosphorus (HP) and low phosphorus (LP) availability at 45 days after planting in greenhouse mesocosms (GH). The data shown are means of 4 replicates of the 4 genotypes in each phenotypic group in either HP or LP ± SE. Different letters represent significant differences ($p \leq 0.05$).

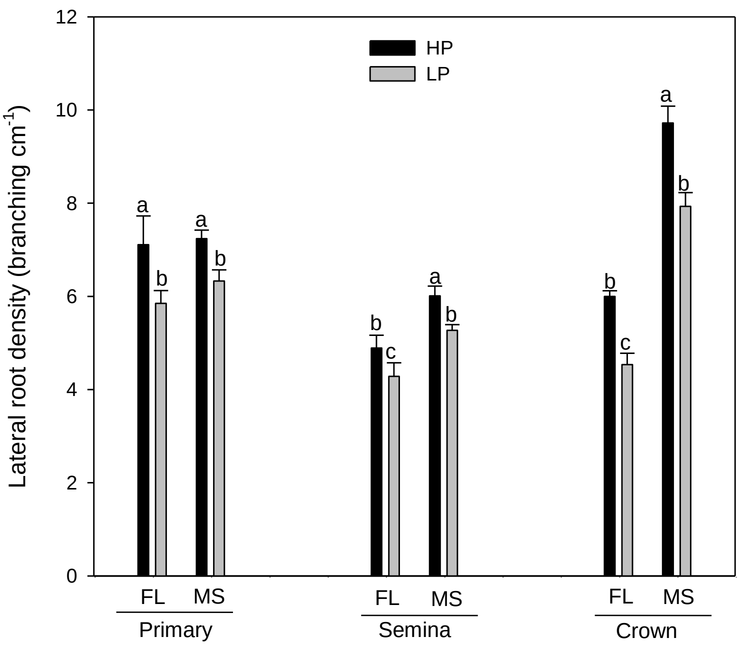

Supplemental Fig. S2 Lateral root branching density of primary root, seminal root and crown root under high P (HP) and low N (LP) availability of MS (many-short) and FL (few-long) lateral root phenotypes at VT stage in the field in 2014. The data shown are means of 4 replicates of the 4 genotypes in each phenotypic group in either HP or LP ± SE. Different letters represent significant differences ($p \leq 0.05$).

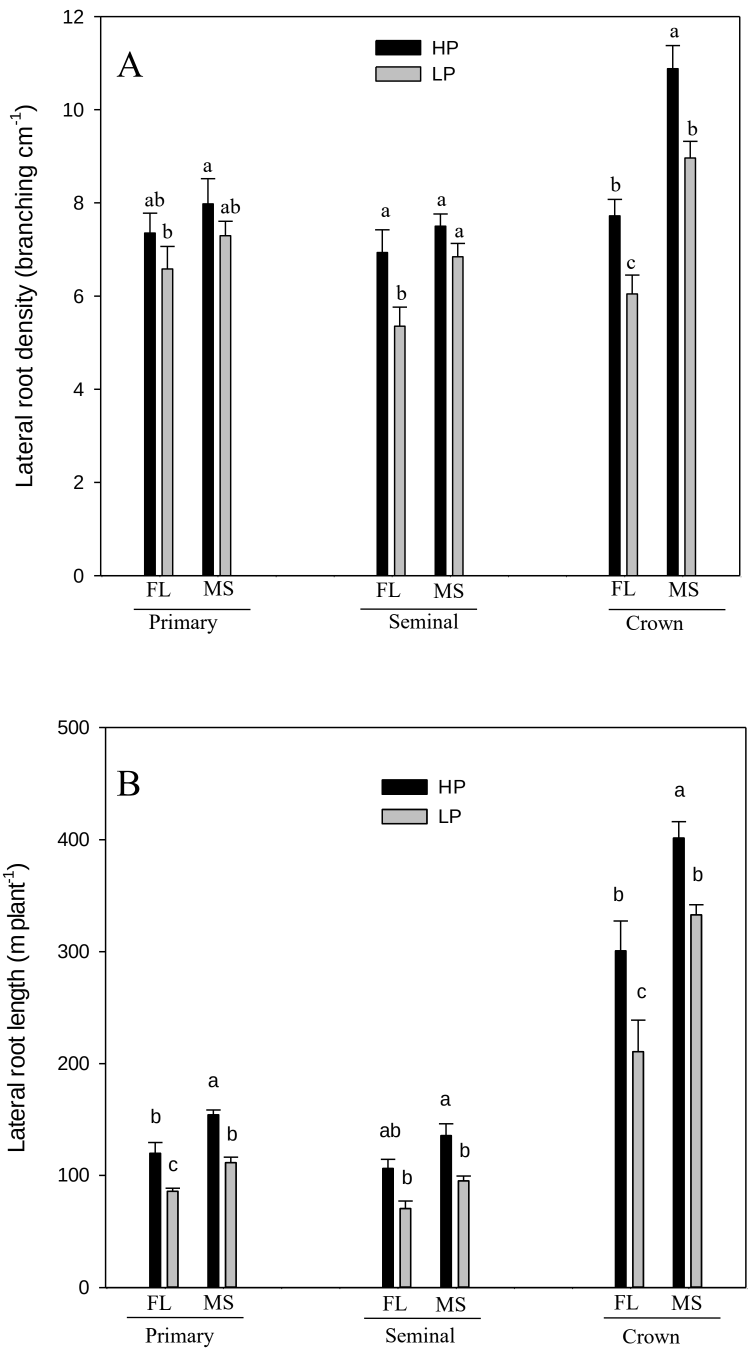

Supplemental Fig. S1 Lateral root branching density (A) and total lateral root length (B) of crown, primary, and seminal root under high P (HP) and low P (LP) availability of MS (many-short) and FL (few-long) lateral root phenotypes at 45 days after planting in greenhouse mesocosms. The data shown are means of 4 replicates of the 4 genotypes in each phenotypic group in either HP or LP ± SE. Different letters represent significant differences ($p \leq 0.05$).
